# Image Guided Intraoperative Radiation Therapy After Surgical Resection of Brain Metastases: A First In-Human Feasibility Report

**DOI:** 10.1016/j.adro.2024.101466

**Published:** 2024-02-05

**Authors:** Molina Grimmer, Gustavo R. Sarria, Motaz Hamed, Mohammed Banat, Fabian Kugel, Hector Lorenzana, Davide Scafa, Mümtaz Köksal, Julian P. Layer, Cas Dejonckheere, Martin Fiebich, Frederic Carsten Schmeel, Ana Kowark, Hartmut Vatter, Leonard Christopher Schmeel, Stephan Garbe

**Affiliations:** a Department of Radiation Oncology, University Hospital Bonn, University of Bonn, Bonn, Germany; b Department of Neurosurgery, University Hospital Bonn, University of Bonn, Bonn, Germany; c Department of Neuroradiology, University Hospital Bonn, University of Bonn, Bonn, Germany; d Department of Neurosurgical Anesthesia, University Hospital Bonn, University of Bonn, Bonn, Germany; e Technische Hochschule Mittelhessen, Gießen, Germany

## Abstract

**Purpose:**

A correct placement of the applicator during intraoperative radiation therapy for brain metastasis is of paramount importance, to deliver a precise and safe treatment. The applicator-to-surface contact assessment cannot be performed under direct observation because the applicator itself limits the visual range. No image guided verification is currently performed intracranially. We hypothesize that image guided intraoperative radiation therapy would assure a more precise delivery in the target area. We describe our workflow in a first in-human experience.

**Methods and Materials:**

Phantom-based measurements were performed to reach the best cone beam computed tomography imaging quality possible. Once defined, a clinical feasibility study was initiated. An in-room cone beam computed tomography device is used to acquire intraoperative images after placing the applicator. Repositioning the applicator is thereafter discussed with the surgeon, according to the imaging outcomes, if required.

**Results:**

An optimal image quality was achieved with 120-kV voltage, 20-mA current, and a tube current time product of 150 mAs. An additional 0.51 mSv patient exposure was calculated for the entire procedure. The wide dynamic range (−600 HU to +600 HU) of cone beam computed tomography and a 27 HU mean computed tomography values difference between brain tissue and spherical applicator allows distinguishing both structures. In this first in-human experience, the applicator was repositioned after evidencing air gaps, assuring full applicator-to-surface contact.

**Conclusions:**

This first in-human procedure confirmed the feasibility of kilovoltage image guided intraoperative radiation therapy in a neurosurgical setting. A prospective study has been initiated and will provide further dosimetric details.

## Introduction

Intraoperative radiation therapy (IORT) presents an alternative or complementary treatment modality for various indications. In the setting of adjuvant irradiation of brain metastases, a spherical applicator is inserted into the surgical cavity and nominal 50-kV x-rays will be delivered to the surface and in limited depth.^1^ Depending on the cavity volume, this single-fraction irradiation allows omitting the standard external-beam treatment, thus reducing hospital visits.^2^^,^^3^ A sharp dose attenuation counts among the features of kilovoltage (kV) irradiation, according to the distance-squared law (equation 1.0).^4^ Because in the kilovoltage spectrum the average range of secondary electrons is rather short, the maximum dose is mostly delivered at the surface. The consequent gradient yields a lower exposure of healthy brain and other surrounding organs at risk.^3^^,^^5^

(1.0) $$I\left(r\right)\;=\;I\left(r_{0}\right)/r^{2}$$

A drawback in IORT is the lack of image-based verification. Positioning the applicator correctly is essential to allow precise dose calculations. This is of utmost importance because the generated x-rays are sharply attenuated over only a short distance range. In practice, the dose is prescribed to the applicator surface (±0.0 mm). This means that neither air gaps nor tissue heterogeneities (eg, hemostatic patches) between the applicator and resection cavity should be present. These could lead to incomplete dose delivery, which are especially relevant in a single-shot procedure,^6^^,^^7^ potentially increasing the risk of local recurrence.^6^^,^^8^ Therefore, without intraoperative imaging, a misplacement cannot be disregarded.^7^^,^^9^

With the implementation of in-room surgical imaging systems, image guided positioning of the spherical applicator has become possible, improving the precision and, thus, therapeutic range of IORT. In addition, this augments the evaluability range of anatomic structures and foreign objects with high x-ray attenuation (eg, bones or surgical instruments), so that possible artifacts are limited and do not hamper the imaging quality. Hence, controlling these factors could minimize any dosimetry-related recurrence risks.^7^^,^^9^

## Methods and Materials

### Implementation of the dosimetric feasibility study

In preparation for the study, 2 simulation procedures were performed at different time points, mimicking an actual surgical scenario, including an interdisciplinary team of neurosurgery, radiation oncology, and anesthesiology.

**Figure 1:**
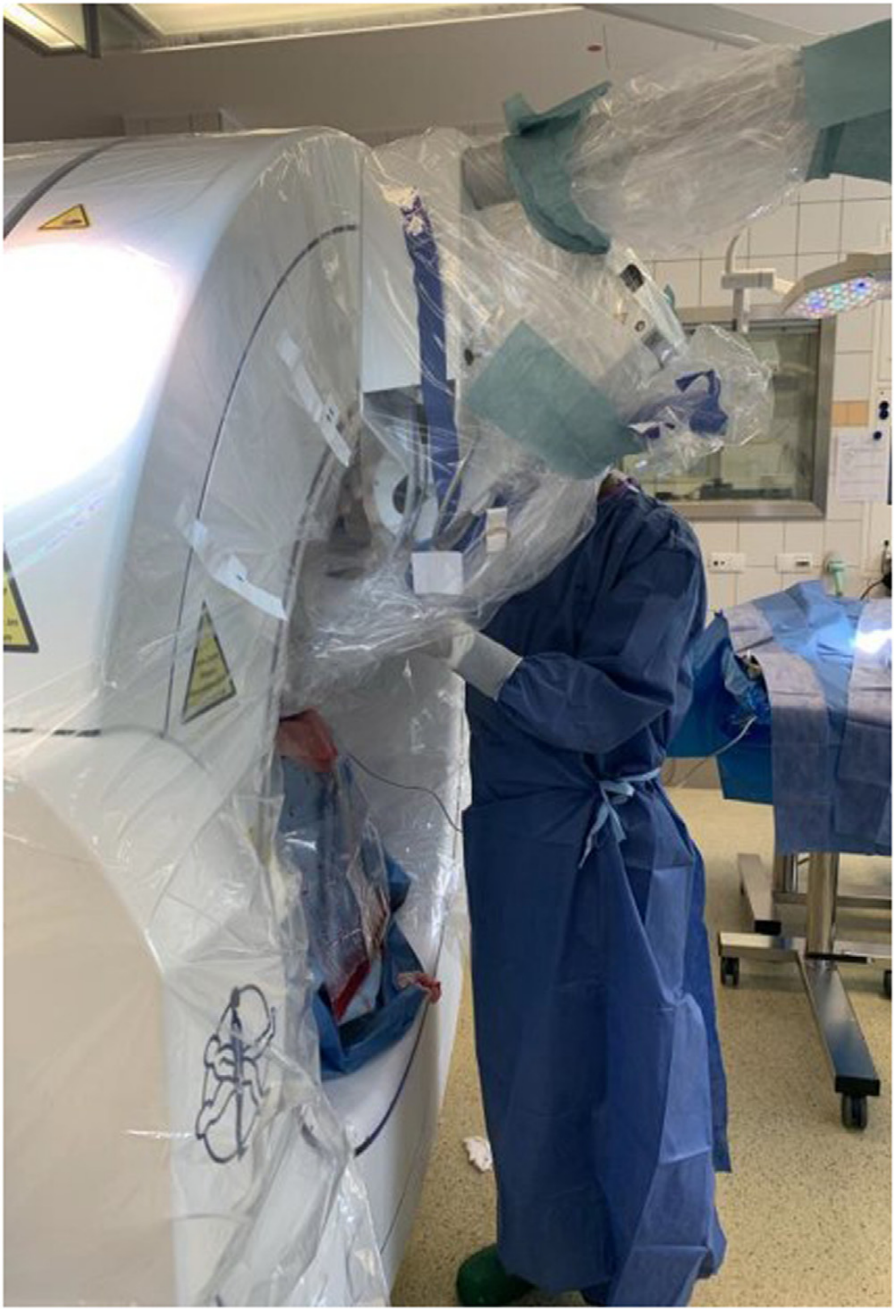
Simultaneous coupling of the in-room cone beam computed tomography kilovoltage intraoperative radiation therapy device.

The focus of the feasibility study was to determine the required image quality of the O-Arm cone beam computed tomography (CBCT; Medtronic Inc) images. The challenge was to ensure the best possible image quality while simultaneously using the INTRABEAM kV-IORT device (Carl Zeiss Meditec AG) and the neurosurgical MRI-navigation StealthStation (Medtronic, Inc). This requires a carbon head mount to avoid artifacts in the CBCT image. Figure 1 shows the compatibility set-up of the in-room CBCT and kV-IORT devices.

**Figure 2:**
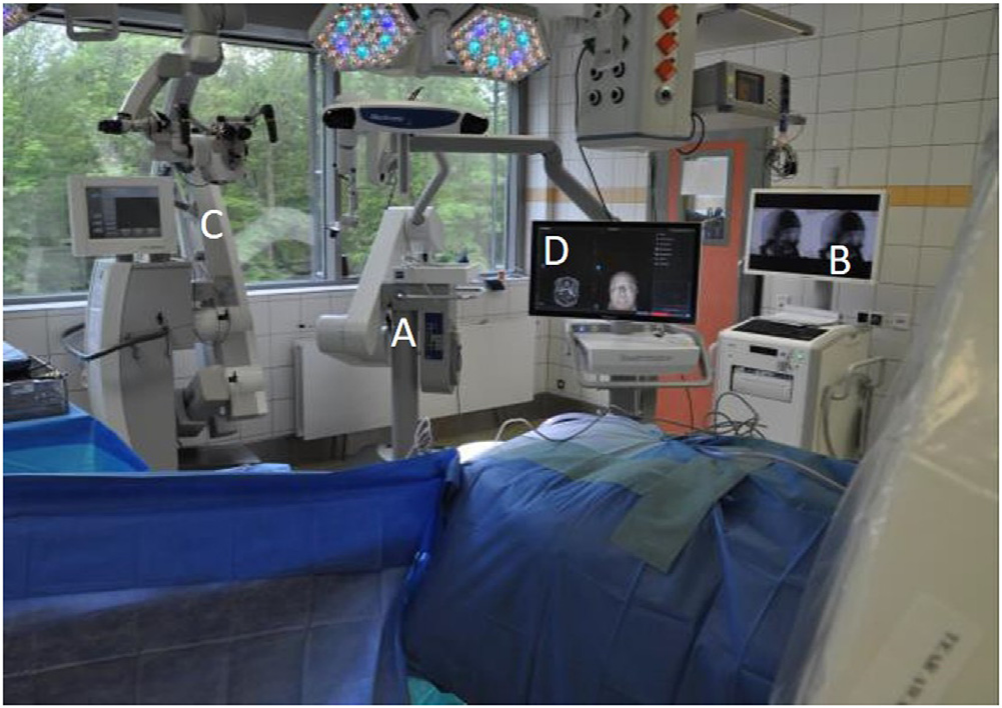
Setup for operating in surgery room. On display are (A) the kilovoltage intraoperative radiation therapy device, (B) cone beam computed tomography mobile display station, (C) the surgical microscope, and (D) the neuronavigation camera and screen.

**Figure 3:**
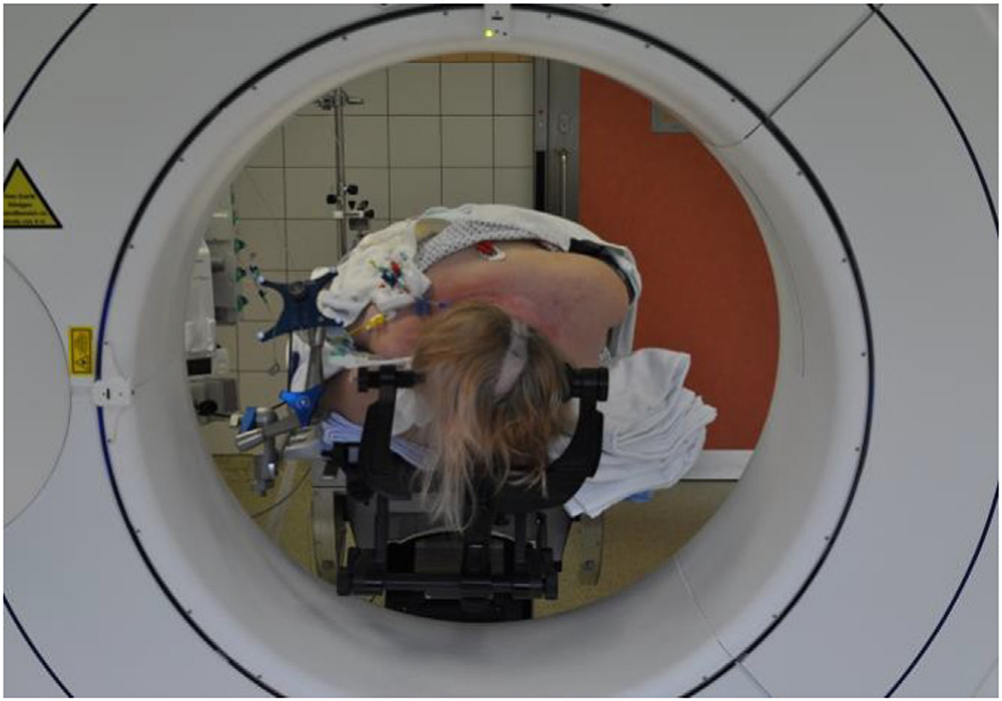
Carbon head mount and neuronavigation stereotactic reference attached to an additional mount. Placement of the patient in the cone beam computed tomography bore.

The neuronavigation stereotactic reference is attached to an additional frame (Fig. 2) on the operating table, distinct from the head support, due to an attachment incompatibility with the carbon head mount. This additional frame allows the patient, the kV-IORT device and the navigation reference to fit within the CBCT bore range (Fig. 2). Noteworthy, the additional frame and navigation reference star include metal parts in their structure; nonetheless, these are far enough for not interfering in the acquisition field. Figure 3 shows the operating room setup, which includes the CBCT, its mobile display station, kV-IORT device, neurosurgical navigation system, operating table, and anesthesia equipment. Additionally, the CBCT will be placed in position before starting surgery, to avoid any shifting that could potentially endanger the patient. For ensuring an adequate imaging quality, all surgical metallic elements are removed from the scan field.

**Figure 4:**
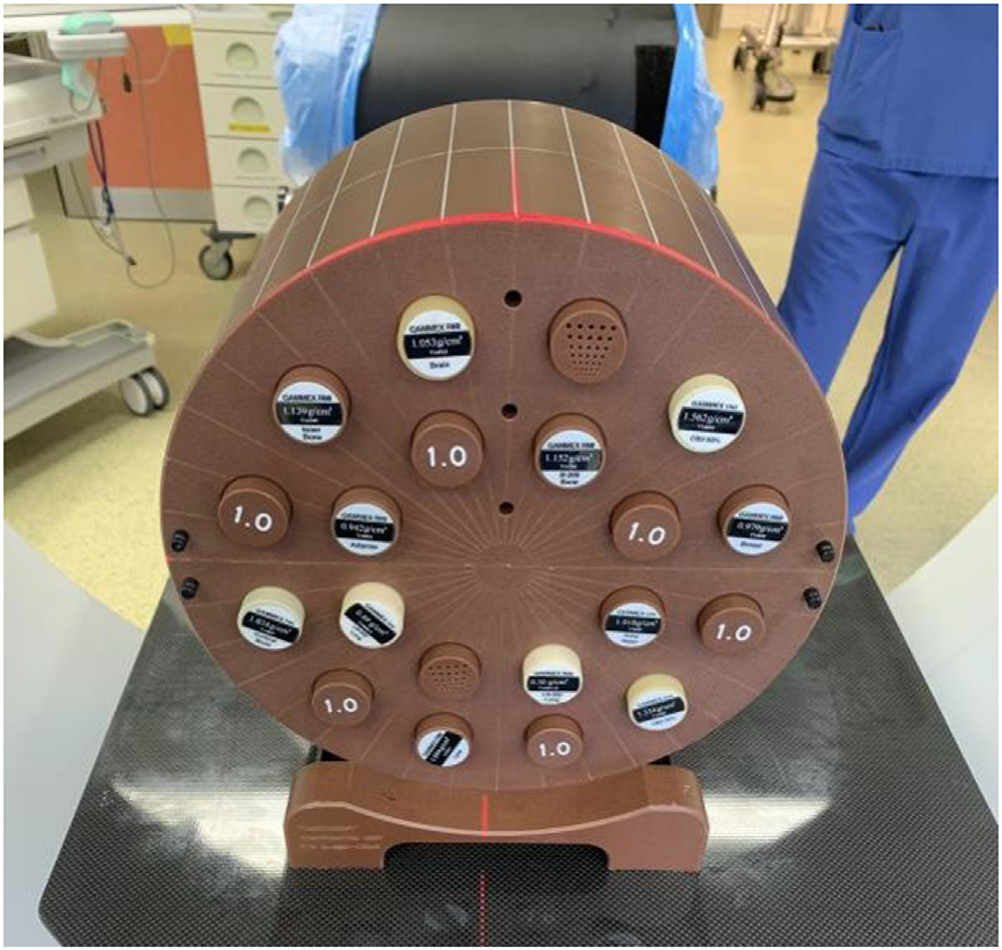
The 12-inserts phantom with various densities for creating an imaging value-to-density table/calibration curve. 1.0 = water equivalent.

To determine the best possible image quality, CBCTs in different imaging modes were acquired with the “cheese phantom” (Gammex Inc; Fig. 4). The images obtained with it are used to generate the imaging value-to-density table/calibration curve (IVDT). Twelve inserts with different density levels ranging from 0.300 g/cm^3^ to 1.842 g/cm^3^ are installed in the phantom and HU (Hounsfield units) values are assigned to each of them. Two inserts have cylindrical cavities with different diameters for assessing spatial resolution. The IVDT is created using image data from the Picture Archiving and Communication System.

### Implementation of the study in the clinical setting

IORT of brain metastases was performed as described in previous studies.^2^, ^3^, ^10^^,^^11^^,^^12^ Study patients are educated regarding the additional effective dose of intraoperative imaging as part of the study. Before IORT is performed, a low-dose fluoroscopy imaging for positioning is acquired. This image ensures that the head is located centrally within the gantry. The table and CBCT position are saved so that the surgeon can adjust the table as needed during surgery. The CBCT acquisition takes place after the brain metastasis has been resected and the spherical applicator has been placed in the cavity. As per the surgeon's criterion, positioning corrections can be considered if the applicator is not completely in contact with the surgical bed or the applicator diameter prevents it. If that is the case, the applicator will be repositioned. A second CBCT is performed after repositioning for confirmation.

## Results

A wide dynamic range of Hounsfield units values are necessary for good differentiation and detectability while maintaining low radiation exposure for the patient. Because the effective dose of the “HD3D large” imaging mode (120 kV, 20 mA, 150 mAs) is 0.51 mSv and due to the large dynamic range in HU values (+600HU to –600HU) of the HD3D, this imaging mode was selected for the study.

**Figure 5:**
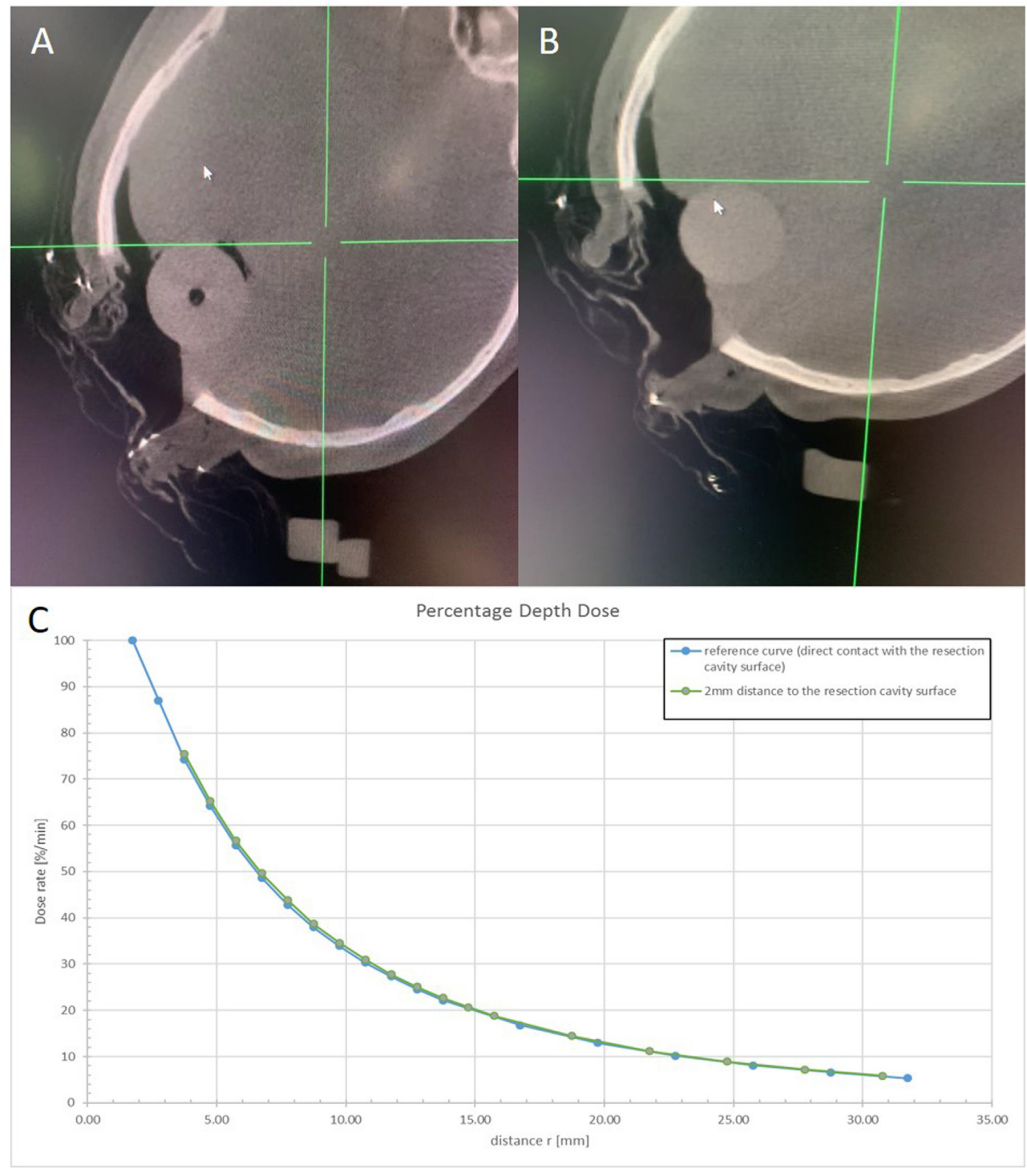
(A) cone beam computed tomography image after positioning the spherical applicator. A 3.5-cm diameter applicator was selected for this case. A 2-mm air gap can be observed between the applicator and brain tissue. (B) Cone beam computed tomography image after repositioning. (C) The blue line depicts a regular dose absorption pattern in case of perfect applicator-surface contact. The green line shows a dose-delivery pattern after a 2-mm air gap, delivering 75% of the prescription dose to the surface.

For distinguishing the spherical applicator from the surrounding tissue (brain density = 1.053 g/cm^3^), its values were obtained directly from the DICOM set and inserted in the IVDT of the HD3D imaging mode. The spherical applicator is made of polyetherimide (ULTEM, Polytron GmbH) and has a density of 1.27 to 1.51 g/cm^3^.^13^ With the HU value determined in the Picture Archiving and Communication System and the HD3D (large)-CBCT, the obtained density was 25 HU, and the mean brain HU-value was −2 HU, allowing a good differentiation profile between both structures (Fig. 5).

Figure 5 shows the resulting images of the “HD3D large” mode. Figure 5A shows the first positioning attempt, and Fig. 5B the correction after an air gap was identified between the applicator and the surgical cavity. Figure 5C depicts the differences among dose-distribution profiles when in contact or with a 2-mm air gap in between, such as in this case. The cavity was irradiated with 30 Gy prescribed to the surface, entailing a treatment time of 21:30 minutes. After irradiation, surgery was completed as usual.

## Discussion

This first in-human experience has evidenced that our proposed workflow is feasible and potentially meaningful for patients. This first attempt required repositioning the applicator toward the resection cavity to minimize the gap with the target tissue (Fig. 5). With additional experience, the entire procedure duration (considering irradiation time) at our institution diminished from 45 minutes to approximately 30 minutes in subsequent patients. Of note, the total time required depends highly on the irradiation time, which depends likewise on the dose (usually 20-30 Gy) and applicator diameter. The additional effective radiation dose for the patient is 0.51 mSv per CBCT. According to our procedure, a maximum of 2 CBCTs per patient are performed in case of correction. The area of interest of the CBCT also represents the irradiation area; therefore, the dose attributable to it could be considered negligible compared with the applied IORT dose (approximately 0.05% ratio). The imaging mode is sufficient for dimensional differentiation between brain and applicator due to the applicator density being markedly greater than that of the surrounding soft tissue.

These outcomes provide new insights for IG-IORT. Nevertheless, certain challenges arise. Live in-room planning is currently not available, as the imaging quality does not allow accurate Monte Carlo calculations with the Radiance system (Radiance, GMV SA). Therefore, only water-based dose estimations can be considered currently. Nevertheless, our team is working on a solution to enable assessing differences pre- and postcorrection. An ongoing prospective study will help elucidate the actual clinic and dosimetric role of IG-IORT in the neurosurgical setting.

## Conclusion

IG-IORT in neurosurgery proved a feasible and practical in this first in-human experience, allowing a more precise positioning assessment before dose delivery. An ongoing prospective study has been initiated and will provide further dosimetric details regarding applicator repositioning.

## Disclosures

Gustavo R. Sarria reports no personal fees and travel expenses from Carl Zeiss Meditec AG, not related to this work; speakers funding from Buro Carl Zeiss Meditec AG. Molina Grimmer reports travel expenses from Carl Zeiss Meditec AG, not related to this work. Hartmut Vatter reports travel expenses from Carl Zeiss Meditec AG, not related to this work.
